# Impact of School-Based Oral Health Education on Vietnamese Adolescents: A 6-Month Study

**DOI:** 10.3390/ijerph18052715

**Published:** 2021-03-08

**Authors:** Vy Thi Nhat Nguyen, Takashi Zaitsu, Akiko Oshiro, Tai Tan Tran, Yen Hoang Thi Nguyen, Yoko Kawaguchi, Jun Aida

**Affiliations:** 1 Department of Oral Health Promotion, Tokyo Medical and Dental University, Tokyo 113-8510, Japan; vy.ohp@tmd.ac.jp (V.T.N.N.); oshiohp@tmd.ac.jp (A.O.); yoko.ohp@tmd.ac.jp (Y.K.); aida.ohp@tmd.ac.jp (J.A.); 2 Faculty of Odonto-Stomatology, Hue University of Medicine and Pharmacy, Hue University, Hue 530000, Vietnam; tttai@huemed-univ.edu.vn (T.T.T.); hoangyenrhm@gmail.com (Y.H.T.N.)

**Keywords:** health education, secondary schools, oral hygiene, adolescent, Vietnam

## Abstract

We have evaluated the impact of a school-based intervention on oral health knowledge, behaviours, and oral health status of adolescents in Vietnam. This 6-month study included 462 adolescents aged 12 years from four selected schools in Hue City, Vietnam. The intervention group received a 15-min lecture by a dentist and hands-on session on mouth observation and toothbrushing skills. The control group did not engage in any educational activities during the follow-up period. Data were collected at baseline and 6 months through a survey questionnaire and clinical examination. The Debris Index was used for dental plaque; the Papillary, Marginal, Attached gingiva index for gingivitis; and the Decayed, Missing, and Filled Teeth index (World Health Organization modification) for dental caries. Difference-in-difference analysis was used to compare changes between the groups. After 6 months, the control tended to show decreased toothbrushing frequency and increased dental plaque accumulation. The participants in the intervention group showed improved oral health knowledge (*p* < 0.01), behavior (*p* < 0.05), and hygiene (*p* < 0.001) compared to the control group. However, the intervention did not improve dental caries and gingivitis. A single school-based oral health education program can help adolescents improve oral health knowledge and prevent the deterioration of short-term oral health behavior and hygiene.

## 1. Introduction

Oral diseases are one of the most prevalent childhood problems and remain a major public health burden worldwide [1,2]. Children with poor oral health are more likely to experience toothache and poor performance at school [3]. Inadequate plaque removal has been shown to increase the risk of tooth decay and gingivitis [4,5]. Consequently, there is broad agreement that oral hygiene behavior is important for everyone and should be performed every day from the beginning of the first tooth eruption [6,7]. Therefore, prevention programs that aim to improve oral hygiene and strengthen oral health in children have been developed in a range of different countries [8]. To our best knowledge, however, there are relatively few studies on adolescents, particularly in low- and lower-middle- income countries. Adolescents, as 12-year-old, have completed their permanent dentition and are independently able to take care of their oral health.

Adolescence is a critical period for health promotion [9]. Evidence has shown that relatively stable patterns of health-related behaviors are established during adolescence and it is difficult to change these behaviors during adulthood [10,11]. To improve oral health, it is necessary to focus on adolescents, as proper personal oral hygiene and eating habits are developed during this stage of life [12]. Evidence indicates that adolescents with favorable oral health habits have better oral health as an adult than those with poorer oral health habits [11]. Consequently, targeting adolescents when promoting oral health can be beneficial [9].

For school-aged children, school-based settings are more common and effective at providing preventive care than a community-based approach [13]. School-based oral health education (OHE) has been applied successfully in some developing countries to achieve better oral health behavior and dental hygiene status of adolescents at a low cost [13,14,15,16,17]. In Vietnam, however, promoting oral health among adolescents has been neglected in public health, and there are few documents reporting the effectiveness of school-based OHE programs in Vietnamese adolescents. An oral health promotion program has been established in Vietnam since the 1980s to reduce the incidence of oral diseases among children [18]. However, this program was implemented only in primary schools, and its effect is insufficient [18,19]. The incidence of oral diseases among Vietnamese children has a tendency to increase with age, and the oral health behavior of school children has not improved [18,19,20]. Therefore, the current study aimed to assess the effectiveness of an OHE program on oral health-related knowledge, behavior, and oral health status of Vietnamese adolescents.

## 2. Study Population and Methodology

### 2.1. Study Design and Sample

This 6-month follow-up study was conducted from March 2019 to September 2019 in Hue City, Vietnam. A cluster sampling method was used to recruit participants. First, Hue city was divided into two areas: urban and suburban area. Next, we obtained a list of all schools from each area, and then two schools were randomly selected from each list. Among the 23 middle schools in Hue City, two were randomly selected in urban and suburban areas, respectively. All the selected schools are public schools with the same curriculum and no experience of OHE. We randomly assigned one urban and one suburban school into the intervention group (IG), and the others were assigned to the control group (CG). At baseline, participants in the IG received one-session of OHE, whereas the CG did not receive any oral health-related educational activities. For ethical considerations, the same OHE at baseline was delivered to the CG after data collection at follow-up. The questionnaire survey and oral examination procedure were conducted at baseline and 6 months.

The sample size was calculated based on a previous study of OHE in 12-year-old students by Solhi et al. [21]. This study stated that oral hygiene improved by 24% in the IG and 12% in the CG. Taking power of 80% at a 5% level of significance, and adjusting by 10% for the non-response rate, a minimum of 173 participants was required for each study arm according to the sample size estimation for two proportions in clinical studies [22].

The target group for the education program was 12-year-old students. This stage is the early period of adolescence, which is important for health education. Also, at the age of 12, children complete the transition from ‘mixed dentition’ into permanent dentition, and it requires more caution on oral care. Participants were all 12-year-old students of middle schools who provided informed consent and had no critical medical problems. Students who were absent at baseline or at any intervention appointment were excluded from this study. The detailed number of participants is described in the results section.

The present study was approved by the Ethics Committees of Hue University of Medicine and Pharmacy, Vietnam, and Tokyo Medical and Dental University, Japan (no. D2018-043). The official permission was obtained from the Department of Education and Training of Hue city and the participating school authorities before conducting the study.

### 2.2. Data Collection Using the Questionnaire

Oral health-related knowledge and behaviors were evaluated using a self-administered questionnaire. The oral health knowledge questionnaire was developed from the oral health knowledge test applied by Blizniuk et al. [23] to validate a version of the oral health literacy instrument. The original version was used for adults, in which some periodontitis-related questions were not suitable for application in children. Therefore, we replaced these items with questions related to common problems among adolescents. The modified questionnaire comprised 10 items concerning: (1) dental caries, (2) gingivitis, (3) dental plaque, (4) toothbrushing habits, (5) dental check-up, (6) relationship of general health and oral health, and (7) impact of smoking on oral health (Appendix A Table A1). Each correct response was awarded 1 point; an incorrect or ‘don’t know’ answer was scored as 0. The cumulative score was calculated for a total oral health knowledge score (0–10). The content validity of the modified oral health knowledge questionnaire was confirmed by the expert panel. Test-retest reliability was confirmed in a sample of 65 subjects using an interval of 3 weeks. The intraclass correlation coefficient for the total score of modified oral health knowledge was 0.87, and Cronbach’s alpha was 0.91.

The oral health behavior questions included four items related to toothbrushing frequency, use of fluoride toothpaste, having a dental visit within 12 months, and sugary food consumption frequency. The desirable behaviors were: (1) brushing teeth at least twice a day, (2) use of fluoride toothpaste when cleaning teeth, (3) visiting the dentist in the last year (for regular check-ups or treatment), and (4) snacking/drinking sugary foods less than once a day. A desirable behavior response was scored as 1 point; otherwise, it was scored as 0. The oral health behavior score (0–4) was calculated by adding the score of each question.

### 2.3. Oral Examination

All students were clinically examined in classrooms by the same two calibrated dentists at baseline and at the 6-month follow-up. The oral cavity was examined using a headlight, dental mirror, and a WHO periodontal probe. Dental caries was assessed using the Decayed, Missing, and Filled Teeth (DMFT) index. Oral hygiene was evaluated using the Debris Index (DI) from Greene and Vermillion’s Oral hygiene index (OHI) [24], and gingival status was assessed using Massler’s Papillary, Marginal, Attached gingiva (PMA) index for 12 anterior teeth [25]. The Kappa coefficient values for the calibration were 0.91, 0.84, and 0.81 for dental caries, DI, and the PMA index, respectively. After examination, each student received a report of their dental caries, gingivitis status, and treatment indication if needed.

### 2.4. Masticatory Performance Test

Masticatory performance was measured using color-changeable gum (Masticatory Performance Evaluating Gum XYLITOL^®^, Lotte Co., Ltd., Saitama, Japan). Participants chewed the gum 60 times for 60 s, and then put it into a plastic bag. The color of the gum was measured according to the procedure described by Kamiyama et al. [26]. Changes in color were assessed using the CIELAB color space defined by the International Commission on Illumination, which specified color by three values: L* for lightness from black (0) to white (100); a* from green (−) to red (+); and b* from blue (−) to yellow (+). The degree of color change before and after chewing, Δ*E*, was determined by the following formula, in which 72.3, −14.9, and 33.0 were the values of L*, a*, and b* before chewing, respectively [27]:

$$∆E=\sqrt{{\left(L^{*}-72.3\right)}^{2}+{\left(a^{*}+14.9\right)}^{2}+{\left(b^{*}-33.0\right)}^{2}}$$

Higher Δ*E* indicates better masticatory performance.

### 2.5. Oral Health Education Intervention

After completing the survey and examination at baseline, OHE was delivered to the IG only. Students received one 45-min session of OHE. The education was facilitated by a dentist, including a short lecture and experiential learning (EL)-based hands-on session on practising mouth observation and tooth brushing. EL is a method in which learning is achieved through experience [28] and has been used effectively in modifying health-related behavior [29,30]. Recently, some EL intervention studies have found that it was more effective in improving oral hygiene than traditional lectures [31,32,33]. In addition, a self-assessment form of dental plaque and gingivitis described in a previous study by Shizuma et al. [34] was used as a supportive tool for learning. The education session started with a 15-min power-point lecture conducted by a dentist in the classroom. The lecture focused on dental plaque formation and characteristics, gingivitis aetiology and symptoms, toothbrushing technique, and the role of a sugary diet in dental caries and gingivitis. After completing the lecture, all students observed their mouths with a hand mirror (PROSPEC dental mirror). Students recorded the dental plaque and gingivitis condition of their anterior teeth on the self-assessment form. They subsequently used a disclosing solution (Hamigaki jōzu PRO, Shofu Inc., Kyoto, Japan) to see dental plaque on their teeth. Students recorded their plaque condition again and compared it with the previous one. After this, each participant received a toothbrush to brush their teeth while using the hand mirror to observe their brushing efficacy. In the hands-on session, the students performed all the activities independently without the educator’s supervision. The dental mirror and toothbrush were given to participants to practice at home, and no further education was provided within the follow-up.

### 2.6. Statistical Analyses

First, to compare the characteristics between CG and IG, independent *t*-tests and Chi-square tests were applied. For evaluating the changes in oral health knowledge, behavior, and status within each group during the follow-up, paired *t*-tests were used. Next, the difference-in-difference (DiD) analysis was used to explore the effect of intervention after 6 months. The DiD estimators measure the intervention effect by comparing the difference of outcomes before and after intervention between the IG and CG [35]. It allows researchers to control the influence of unobserved variables that affect both the intervention and the control identically [36]. To detect the patterns of changes in multiple outcomes, we performed a series of linear regression analyses using the following equation [37]:

Yi = β0 + β1 × Time + β2 × Intervention + β3 × (Time × Intervention) + β4 × covariates + εi

β0, β1, β2, β3, and β4 are the coefficient of baseline average, time trend in the control group, the difference between the two groups pre-intervention, the difference in changes over time, and covariates, respectively. εi is the error terms.

Yi is the outcome variable for each student: clinical oral health (dental caries, DI score, and PMA score), oral health knowledge, and oral health behavior. The intervention defined ‘control group’ as 0 and ‘intervention group’ as 1. Time defined ‘baseline’ as 0 and ‘6-month follow-up’ as 1. ‘Time x Intervention’ was also included as the independent variable. Each regression was adjusted for all socio-demographic covariates. These models were built for each outcome.

Data were analysed using the Statistical Package for Social Sciences (IBM SPSS version 21.0; IBM Co., Armonk, NY, USA). The level of statistical significance for all tests was set at *p* < 0.05. The paper is reported following the JBI Critical Appraisal Checklist for Quasi-Experimental Studies [38].

## 3. Results

### 3.1. Descriptive Statistics

**Figure 1:**
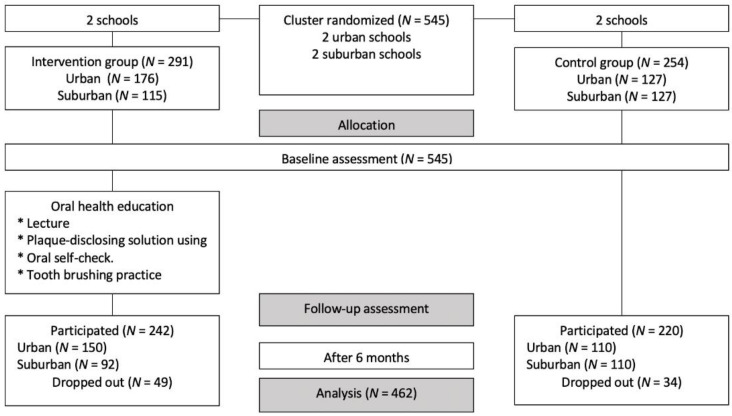
Study flowchart.

Initially, 545 students in four schools were allocated to either the IG (*n* = 291) or the CG (*n* = 254) (Figure 1). The dropout rate at the 6-month follow-up was 16.8% and 13.4% for the IG and CG, respectively. The final analysis included 242 participants in the IG and 220 in the CG. The sociodemographic characteristics of participants in the two groups are presented in Table 1. The groups had significant differences in all socio-demographic characteristics, except for the mother’s level of education.

#### 3.1.1. Oral Health-Related Knowledge

At baseline, the oral health knowledge score in the IG was significantly higher than that in the CG (Table 2). However, both groups showed lower scores related to knowledge of dental plaque and gingivitis when compared to knowledge related to dental caries (Q1, Q2), toothbrushing habits (Q7), and dental visit habits (Q8) (Appendix A Table A1). From baseline to the 6-month follow-up, the mean score of oral health knowledge significantly increased only in the IG (Table 2). In detail, 6 months after the intervention, the IG had significantly improved scores in Q1–Q2 (related to dental caries), Q3–Q4 (related to gingivitis), Q6 (‘use of fluoride makes teeth stronger’), and Q10 (‘smoking can cause oral cancer’). In contrast, the CG had a significantly decreased score in Q7 (‘the teeth should be brushed at least twice a day’) and had no improvement in the scores of the other questions (Appendix A Table A1).

#### 3.1.2. Oral Health-Related Behaviors

The over-time changes in the oral health behavior of the two groups are described in Table 2. Before the intervention, oral health behavior scores were not significantly different between the groups. At 6 months post-intervention, only the IG showed a significant improvement in oral health behavior score. The comparison between the two groups also showed that the behavior score at follow-up was significantly higher in the IG than in the CG. Specifically, the ‘brushing teeth twice a day’ and ‘using fluoride toothpaste’ habits showed significantly higher scores in the IG than in the CG at the 6-month follow-up.

#### 3.1.3. Oral Health Status

The results of the clinical examination and chewing test are presented in Table 2. At baseline, no significant difference in the oral health status existed in either group, except for the gingival health index (PMA score), which was significantly higher in the CG than in the IG. At 6 months post-intervention, the number of decayed teeth and the PMA score were significantly increased in both the IG and CG. Oral hygiene (DI score) worsened in the CG, but no change was found in the IG. According to the chewing test results, however, masticatory performance significantly improved in both the IG and CG.

### 3.2. Difference-in-Difference Model

Table 3 shows the difference in changing oral health knowledge, behavior, and clinical status in the IG and CG over 6 months. Both the knowledge score and behavior score of the IG were significantly increased when compared with those of the CG (the coefficients were 0.913 and 0.316, respectively). Among the oral health behaviors, there was a relative increase of 0.121 points (*p* = 0.040) in ‘brushing teeth at least twice a day’, and 0.223 points (*p* < 0.001) in ‘using fluoride toothpaste’ in the IG compared to the CG. There were no significant differences in changes in snacking/drinking sugary foods and dental visit experiences between the two groups. The clinical outcomes in the IG, relative to the CG, showed significant improvement only in oral hygiene (DI score decreased by 0.52 points, *p* < 0.001). Changes in the number of decayed teeth, gingivitis status (PMA score), and masticatory performance were not significantly related to the intervention.

## 4. Discussion

This study demonstrated that a simple school-based oral health teaching program, such as the one described here, was able to improve oral health knowledge and behaviour, and had a positive impact on dental plaque control among 12-year-old students. To the best of our knowledge, this is the first attempted intervention in Vietnam to explore the effects of OHE on oral health-related knowledge, behaviors, and status among adolescents.

In the present study, OHE improved knowledge and behaviors, but did not significantly affect the progression of dental caries and gingival status within 6 months, which is consistent with previous OHE studies in school settings. Our results are in line with those of Worthington et al. [39], D’Cruze et al. [17], and Haque et al. [13], which reported the improvement of toothbrushing behaviour and oral hygiene as a short-term effect of OHE. However, the present OHE did not prevent the development of oral diseases. This result is also consistent with countries that conducted similar OHE programs such as Denmark [40], China [14], Greece [33], and Pakistan [41], which demonstrated limited impact on preventing oral diseases. A literature review by Kay and Locker [42] on oral health promotion programs noted that a simple provision of information was sufficient to increase oral health knowledge; however, changes in knowledge, attitude, and beliefs may not lead to a change in behavior or health. As a result, an increase in dental caries was reported in most of the OHE-only interventions [43,44], although changes in gingival status varied between studies. In a review of the effect of OHE on oral hygiene and gingival health in adolescents, Brukiene and Aleksejuniene [9] reported that plaque scores were significantly reduced after behavioral intervention. However, studies addressing the gingival status have reported a broader range of effects; some showed significant improvement in the gingival index score after the intervention, whereas others reported a weak or no effect on gingivitis prevention. These findings suggest that the improvement in oral health knowledge, toothbrushing behavior, and oral hygiene after education is insufficient to affect the oral health status of the participants.

The current study did not reveal any difference in changing behaviors related to daily sugary food consumption and dental visits. Previous studies have reported similar results in sugary food consumption habits, but showed improvements in dental visit practice [14,45,46,47]. In the current study, environmental barriers such as the abundance of sugary snacks and drinks in the school canteen and busy academic schedules may have prevented students from adopting favourable behaviors. Interestingly, even though dental caries and gingivitis worsened, the masticatory performance of the participants improved in both groups. A possible explanation for this is that the DMFT and PMA indices, which were used to measure dental caries and gingivitis outcomes, respectively, express the number of affected units, and not the severity of the diseases.

This study has some implications. Along with general education, a possible factor contributing to our positive results is the oral self-check training using a self-assessment form. Self-assessment (self-diagnosed) of dental plaque and gingival status has been studied since the 1960s in adult patients and has recently been successfully applied to adolescents in reducing dental plaque and gingivitis [48]. In our OHE program, by recording their teeth and gum condition, students could recognise not only their oral hygiene and gingival health, but also the abundance of plaque accumulation in the cervical and interproximal areas of the teeth, which is the most detrimental to their oral health. In addition, our intervention was easy to implement, low-cost, and not time- or labour-intensive. School-based OHE programs conducted in secondary schools face greater difficulties than in those in primary schools. Secondary schools follow a busy curriculum that may make it challenging to incorporate health promotion activities. Adolescence is also considered as the most difficult stage for health educational activities [9]. However, our simple intervention can easily be applied in Vietnamese secondary schools without affecting the students’ school activities. Moreover, universal secondary education has been available in Vietnam for more than 10 years. All Vietnamese adolescents are encouraged to go to secondary school at no cost, which means that socio-economic inequality is not a barrier for adolescents to participate in this program. Therefore, the current OHE program can be a reference model for oral health promotion programs targeting Vietnamese adolescents in the future.

The strength of this study is the use of DiD analysis. The DiD approach provides a powerful method to compare divergent samples when a simple pre- and post-treatment comparison or other risk-adjustment methods may not be sufficient [49,50]. Moreover, we believe that using a wide range of outcomes, including oral health-related knowledge, behaviors, oral hygiene, oral disease, and chewing function, enabled us to comprehensively evaluate the intervention. However, the current study has several limitations that must be noted. First, the sample was selected from Hue city, and thus does not represent the Vietnamese population. Therefore, the present results may not be generalisable to other areas. Second, a high dropout rate may have influenced the results of the intervention. There was a possibility that students with high awareness of oral health tended to participate in the study. If so, our results may be overestimated. Third, although we randomly selected intervention and non-intervention schools, the characteristics of the students in the groups were different. The IG included a higher number of females and students who live in an urban area. Furthermore, the baseline scores of oral health knowledge and PMA were higher in the IG compared to CG; therefore, the effect of our intervention could be overestimated. To reduce the effect of this bias, we conducted the DiD analysis adjusted for all of the socio-demographic variables. Further studies using randomized intervention or cluster randomization design are required. Fourth, the outcome trends of the two groups before the intervention were not known, while the parallel trend is a critical assumption in DiD analysis [50]. This bias could over- or underestimate the effects of the education program. Finally, the long-term sustainable effects of the program remain unknown.

## 5. Conclusions

In conclusion, the school-based OHE in the current study was effective in improving oral health knowledge and preventing the deterioration of toothbrushing behaviors as well as oral hygiene in adolescents. Since this only-education intervention showed no effect on improving gingival health and dental caries status, additional support methods should address in future interventions to overcome this limitation.

## Figures and Tables

**Table 1:** Socio-demographic characteristics of participants.

Variables	Total Number (%)	Intervention Group Number (%)	Control Group Number (%)	*p*-Value ^1^
Total	462 (100)	242 (52.4)	220 (47.6)	
Sex				
Male	210 (45.5)	93 (38.4)	117 (53.2)	0.001
Female	252 (54.5)	149 (61.6)	103 (46.8	
Residency location				
Urban	260 (56.3)	150 (62.0)	110 (50.0)	0.010
Suburban	202 (43.7)	92 (38.0)	110 (50.0)	
Mother’s level of education				
Low (up to secondary school)	160 (34.6)	72 (29.8)	88 (44.0)	0.055
High (High school/College/University)	99 (21.5)	53 (21.9)	46 (20.9)	
Unknown	203 (43.9)	117 (48.3)	86 (39.1)	
Father’s level of education				
Low (up to secondary school)	151 (32.7)	65 (26.9)	86 (39.1)	0.015
High (High school/College/University)	116 (25.1)	63 (26.0)	53 (24.1)	
Unknown	195 (42.2)	114 (47.1)	81 (36.8)	
Mother’s occupation				
Government/Company worker	84 (18.2)	47 (19.4)	37 (16.8)	0.027
Self-employed/freelancer	317 (68.6)	173 (71.5)	144 (65.5)	
Unemployed	33 (7.1)	11 (4.5)	22 (10.0)	
No mother in household	20 (4.3)	10 (4.1)	10 (4.5)	
Unknown	8 (1.7)	1 (0.4)	7 (3.2)	
Father’s occupation				
Government/Company worker	107 (23.2)	64 (26.4)	43 (19.5)	0.007
Self-employed/freelancer	294 (63.6)	159 (65.7)	135 (61.4)	
Unemployed	3 (0.6)	1 (0.4)	2 (0.9)	
No father in household	39 (8.4)	11 (4.5)	28 (12.7)	
Unknown	19 (4.1)	7 (2.9)	12 (5.5)	

^1^ Chi-square test.

**Table 2:** Oral health knowledge, behaviors, and clinical status in intervention and control groups, at baseline and at follow-up.

Variables	Intervention Group			Control Group			*p*-Value ^2^	
	Baseline	Follow-Up	*p*-Value ^1^	Baseline	Follow-Up	*p*-Value ^1^	Baseline	Follow-Up
	Mean (SD)	Mean (SD)		Mean (SD)	Mean (SD)			
**Oral health knowledge**	5.77 (2.19)	6.64 (2.53)	<0.001	5.27 (1.97)	5.22 (2.38)	0.072	0.010	<0.001
**Oral health behaviors**								
Brush teeth twice a day or more	0.76 (0.46)	0.78 (0.42)	0.504	0.70 (0.46)	0.60 (0.49)	0.003	0.174	<0.001
Use fluoride toothpaste	0.20 (0.40)	0.43 (0.50)	<0.001	0.30 (0.46)	0.40 (0.46)	1.000	0.013	0.009
Visit dentist in the past	0.33 (0.47)	0.28 (0.45)	0.175	0.30 (0.46)	0.31 (0.46)	1.000	0.549	0.508
Eat/drink sugar less than once a day	0.35 (0.48)	0.36 (0.48)	0.905	0.39 (0.49)	0.37 (0.49)	0.712	0.330	0.669
Total score	1.64 (0.92)	1.84 (1.00	0.003	1.70 (0.95)	1.59 (1.03)	0.094	0.750	0.008
**Oral health status**								
Number of teeth	26.84 (1.91)	27.34 (1.38)	<0.001	26.39 (2.34)	27.08 (1.74)	<0.001	0.023	0.068
Decayed teeth	2.89 (2.86)	3.38 (2.79)	<0.001	2.61 (2.65)	3.11 (2.76)	<0.001	0.286	0.287
Debris index	2.55 (0.96)	2.53 (1.01)	0.849	2.55 (0.99)	3.07 (0.78)	<0.001	0.834	<0.001
PMA score	14.12 (6.12)	14.98 (5.26)	0.005	16.07 (4.83)	16.83 (4.57)	<0.001	<0.001	<0.001
Masticatory performance (Delta E)	47.1 (6.1)	50.0 (5.8)	<0.001	46.9 (6.4)	48.5 (6.29)	<0.001	0.723	0.009

SD: standard deviation. PMA: papillary, marginal, attached gingiva. ^1^ pair *t*-test. ^2^ independent *t*-test.

**Table 3:** Adjusted difference-in-difference estimates of the impact of oral health education on oral health knowledge, behavior, and clinical status.

Variables	β (SE) ^1^	*p*-Value
**Oral health knowledge**	0.913 (0.300)	0.002
**Oral health behaviors**		
Brush teeth twice a day or more	0.121 (0.059)	0.040
Use fluoride toothpaste	0.223 (0.060)	<0.001
Visit dentist in the past	−0.054 (0.061)	0.373
Eat/drink sugar less than once a day	−0.260 (0.063)	0.676
Total score	0.316 (0.127)	0.013
**Oral health status**		
Decayed teeth	<0.001 (0.353)	0.999
Debris index	−0.517 (0.123)	<0.001
PMA score	0.100 (0.690)	0.884
Masticatory performance (Delta E)	1.274 (0.799)	0.111

β: coefficient, SE: standard error, PMA: papillary, marginal, attached gingiva. ^1^ Sex, residency location, mother’s/father’s level of education, mother’s/father’s occupation and number of teeth were adjusted.

## Data Availability

The data are not publicly available due to privacy and ethical restrictions.

## Appendix A

**Table A1:** Oral health knowledge questions with scores at baseline and 6-month follow-up.

Questionnaire Items	Intervention Group			Control Group			*p*-Value ^2^	
	Baseline	Follow-Up	*p*-Value ^1^	Baseline	Follow-Up	*p*-Value ^1^	Baseline	Follow-Up
	Mean (SD)	Mean (SD)		Mean (SD)	Mean (SD)			
Oral health knowledge total score	5.77 (2.19)	6.64 (2.53)	<0.001	5.27 (1.97)	5.22 (2.38)	0.072	0.010	<0.001
Q1. Dental caries is caused by bacteria of the oral cavity.	0.73 (0.44)	0.83 (0.42)	0.001	0.74 (0.44)	0.73 (0.45)	0.706	0.817	0.005
Q2. Sweet food and drink have positive effects on health.	0.68 (0.47)	0.77 (0.42)	0.011	0.66 (0.48)	0.72 (0.45)	0.134	0.672	0.179
Q3. Bleeding when brushing is a primary sign of gingivitis.	0.46 (0.50)	0.66 (0.47)	<0.001	0.39 (0.49)	0.39 (0.49)	1.000	0.081	<0.001
Q4. Gingivitis is unavoidable	0.47 (0.50)	0.59 (0.49)	0.006	0.32 (0.47)	0.33 (0.47)	0.911	0.001	<0.001
Q5. Plaque is black staining on teeth	0.15 (0.35)	0.18 (0.39)	0.239	0.14 (0.35)	0.10 (0.29)	0.105	0.811	0.008
Q6. Use of fluoride makes teeth stronger	0.51 (0.50)	0.76 (0.43)	<0.001	0.43 (0.50)	0.51 (0.50)	0.059	0.069	<0.001
Q7. The teeth should be brushed at least twice a day	0.92 (0.28)	0.88 (0.33)	0.068	0.94 (0.25)	0.79 (0.41)	0.902	0.436	0.015
Q8. Visit a dentist once a year helps to preserve oral health	0.80 (0.40)	0.79 (0.41)	0.786	0.77 (0.42)	0.76 (0.43)	0.902	0.446	0.456
Q9. Oral health cannot affect general health	0.59 (0.49)	0.63 (0.48)	0.324	0.54 (0.50)	0.48 (0.50)	0.202	0.238	0.001
Q10. Smoking can cause oral cancer	0.45 (0.50)	0.57 (0.50)	0.001	0.35 (0.48)	0.43 (0.50)	0.059	0.022	0.002

Abbreviation: SD, standard deviation. ^1^ pair t-test. ^2^ independent *t*-test.
